# Temporal trends in, and risk factors for, HIV seroconversion among female sex workers accessing Zimbabwe’s national sex worker programme, 2009–19: a retrospective cohort analysis of routinely collected HIV testing data

**DOI:** 10.1016/S2352-3018(23)00110-8

**Published:** 2023-06-14

**Authors:** Harriet S Jones, Bernadette Hensen, Sithembile Musemburi, Lilian Chinyanganya, Albert Takaruza, Sungai T Chabata, Primrose Matambanadzo, Lucy Platt, Brian Rice, Frances M Cowan, James R Hargreaves

**Affiliations:** Faculty of Public Health and Policy, London School of Hygiene & Tropical Medicine, London, UK; Sexual and Reproductive Health Group, Department of Public Health, Institute of Tropical Medicine, Antwerp, Belgium; Centre for Sexual Health and HIV/AIDS Research Zimbabwe, Harare, Zimbabwe; Faculty of Public Health and Policy, London School of Hygiene & Tropical Medicine, London, UK; University of Sheffield, School of Health and Related Research; Centre for Sexual Health and HIV/AIDS Research Zimbabwe, Harare, Zimbabwe; Faculty of Global Health, Liverpool School of Tropical Medicine, Liverpool, UK; Faculty of Public Health and Policy, London School of Hygiene & Tropical Medicine, London, UK

## Abstract

**Background:**

The frequency of new HIV infections among female sex workers in sub-Saharan Africa is poorly understood. We used routinely collected data that enable unique identification of repeat HIV testers to assess temporal trends in seroconversion and identify associated risk factors for female sex workers accessing Sisters with a Voice, Zimbabwe’s national sex worker programme.

**Methods:**

We pooled HIV testing data gathered between Sept 15, 2009, and Dec 31, 2019, from 36 Sisters programme sites in Zimbabwe. We included female sex workers aged 16 years or older with an HIV-negative test and at least one subsequent programme test. We calculated HIV seroconversion rates (using the midpoint between the HIV-positive test and the last negative test as the seroconversion date) and estimated rate ratios to compare 2-year periods by using Poisson regression, with robust SEs to account for clustering by site and adjusting for age and testing frequency to assess temporal trends. We did sensitivity analyses to explore assumptions about seroconversion dates and the effects of variation in follow-up time on our conclusions.

**Findings:**

Our analysis included data for 6665 female sex workers, 441 (7%) of whom seroconverted. The overall seroconversion rate was 3·8 (95% CI 3·4–4·2) per 100 person-years at risk. Seroconversion rates fell with time since first negative HIV test. After adjustment, there was evidence of a decrease in seroconversion rates from 2009 to 2019 (p=0·0053). In adjusted analyses, being younger than 25 years, and having a sexually transmitted infection diagnosis at a previous visit, were significantly associated with increased seroconversion rates. Our findings were mostly robust to sensitivity analyses, but when 1 month before an HIV-positive test was used as the seroconversion date, seroconversion rates no longer fell with time.

**Interpretation:**

We identified high rates of seroconversion shortly after linkage to programme services, which emphasises the need to strengthen HIV prevention programmes from first contact with female sex workers in Zimbabwe. New infections among female sex workers remain challenging to measure, but longitudinal analysis of routine testing data can provide valuable insights into seroconversion rates and associated risk factors.

**Funding:**

UN Population Fund, Deutsche Gesellschaft für Internationale Zusammenarbeit, the Bill & Melinda Gates Foundation, The Global Fund to Fight AIDS, Tuberculosis and Malaria, US President’s Emergency Plan for AIDS Relief, US Agency for International Development, and the Elton John AIDS Foundation.

## Introduction

Understanding trends in new HIV infections and risk factors for seroconversion is essential for the optimisation of programmes aiming to sustainably control the epidemic.^1^ The number of new HIV infections has plateaued at 1·5 million globally, with 58% of these infections in sub-Saharan Africa.^2^ A focus on preventing new HIV infections among key populations rather than more broadly in lower risk networks will have a larger effect on overall HIV transmission.^3,4^ Female sex workers are one such key population: they bear a disproportionally high burden of HIV,^5^ and have a 30 times greater risk of infection than non-sex-working women of reproductive age.^2^ Female sex workers indirectly accounted for an estimated 15% of new infections in sub-Saharan Africa in 2021.^2^

Female sex workers are often mobile, transient, stigmatised, and criminalised,^6,7^ which creates barriers to accessing HIV services^8^ and difficulties in recruiting and following up cohorts over time. As a result, measurement of new HIV infections in this population is challenging. Uncertainty around population size and challenges with reaching female sex workers have prompted the use of alternative methods, such as network sampling or respondent-driven sampling.^6,9^ Despite these advances, data for new HIV infections among female sex workers in sub-Saharan africa and temporal trends are poorly understood.^10,11^ Leveraging routinely collected data among repeat testers in which each person is assigned a unique identifier could provide an opportunity to explore HIV seroconversion and associated risks among female sex workers accessing HIV testing services over time, and provide insights not obtained by other approaches.

In Zimbabwe, the prevalence of HIV among female sex workers is estimated to be 57·5%.^12^ HIV testing data from Zimbabwe’s national sex work programme, Sisters with a Voice (referred to hereafter as Sisters), provides a unique opportunity to understand trends in HIV seroconversion in a programme context. We aimed to understand trends in HIV seroconversion among women who underwent repeat testing within the Sisters programme between 2009 and 2019, to identify risk factors associated with seroconversion, and to assess and minimise potential biases.

## Methods

### Study setting and data sources

We did a retrospective cohort analysis of HIV testing data routinely collected by Sisters, a national sexual and reproductive health programme in Zimbabwe that provides free services mainly to cisgender women and girls aged at least 16 years who self-identify as a sex worker. Between the programme’s intiation in September, 2009, and Dec 31, 2019, Sisters operated at 36 sites, including static clinics delivering services 5 days a week and mobile clinics delivering services once a week. Community outreach is done at each site by peer educators. HIV testing is offered at the first clinic visit to woman who are HIV negative or of unknown HIV status. HIV-negative women revisiting any Sisters clinic are offered an HIV test if they have not been tested within the previous 6 months, in line with national guidance. Zimbabwe’s national HIV testing algorithm is followed at all Sisters clinics, with Determine HIV-1/2 antibody testing (Abbott Diagnostics, Tokyo, Japan) for initial screening and confirmatory antibody testing with SD Bioline HIV-1/2 (Abbott Diagnostics, Tokyo, Japan). Until 2018, female sex workers who tested positive for HIV were referred for treatment at Government health facilities, in line with national treatment guidelines at the time of diagnosis. Antiretroviral therapy (ART) is now provided by Sisters clinics in 11 districts.

A unique identifier code and a Sisters number are assigned to each woman when they first engage with the programme, either at the first clinic visit or during community outreach. Personal identifying information, including phone numbers and current location, are collected by clinic staff during registration and kept electronically. At subsequent visits, women are identified by their Sisters number or unique identifying information. To minimise duplicate records (ie, the assignment of more than one Sisters number to the same individual), checks of personal identifying information provided at registration are done by clinic staff. At the first clinic visit, staff also collect data for age, marital status (married, divorced, never married, separated, or widowed), education (none or primary education, or secondary or tertiary education), and whether the client has ever experienced gender-based violence. At each clinic visit, data are collected for self-reported HIV testing history and sexual risk behaviour, including condom use with most recent sex partner. A record is kept of services provided at each visit, including HIV test results and diagnosis of sexually transmitted infections (STIs; based on verbal report of symptoms and a physical examination only).

Ethical approval for our study of Sisters data was obtained from the London School of Hygiene & Tropical Medicine (16543) and the Medical Research Council of Zimbabwe (MRCZ/A/2624). Because the data were collected as part of routine clinical care, consent was not obtained. Data were de-identified and anonymised before databases were shared for analysis.

### Procedures

To study seroconversion rates, we formed a retrospective cohort comprising women visiting Sisters who had an initial HIV-negative test and who underwent one or more subsequent HIV tests as part of the programme between Sept 15, 2009, and Dec 31, 2019. We excluded women with fewer than 31 days between their first and last HIV test (because of the minimal follow and the possibility that those testing HIV positive could have been seroconverting at the time of their first test). We excluded HIV tests that were done with 7 days of a previous test and people who tested HIV negative after a previous positive test result, because we could not guarantee the accuracy of these data. Finally, to ensure equal opportunity for at least 1 year’s follow-up and to prevent artificially inflating seroconversion rates by limiting the potential to return for subsequent testing,^13^ we excluded women whose first HIV test was after Dec 31, 2018. Cohort entry was the date of a woman’s first HIV test at a Sisters clinic. Cohort exit was either an estimated seroconversion date or the date of the last HIV-negative test. The date of seroconversion was estimated as the midpoint between a woman’s last negative HIV test and her HIV-positive test.

Our main exposure was time. We used lexis expansion to split data into 2-year periods (2009–11 [slightly longer than 2 years because it also contained data for the end of 2009], 2012–13, 2014–15, 2016–17, and 2018–19) and by time since first HIV test (<6 months, 6 to <12 months, 12 to <18 months, and ≥18 months). Other explanatory variables included demographic and behavioural factors, HIV testing, and clinic visit characteristics—ie, the results of any other assessment at the clinic, such as syndromic STI diagnoses, and the location (urban *vs* rural) and type (mobile *vs* static) of clinic. We calculated individual HIV testing frequency by using the mean time between a woman’s Sisters tests (<6 months, 6 to <12 months, 12 to <18 months, and ≥18 months). Testing frequency was not treated as time-varying and was thus calculated once for the duration of individual follow up. Time-varying factors were collected at each visit (except for age, which was calculated on the basis of data collected at the first visit). We used lexis expansion to split data on age at HIV test for age (<25 years and ≥25 years). Other variables were not treated as time-varying, because data were collected at first visit only, or there was minimal individual-level variation over time (eg, clinic site).

### Statistical analysis

We described our cohort by HIV test and clinic visit characteristics and demographic and behavioural risk factors, and stratified data by whether or not participants tested positive for HIV. We described these characteristics for the whole analysis period (ie, 2009–19) and then for 2009–11, 2012–13, 2014–15, 2016–17, and 2018–19. We calculated seroconversion rates overall and then by study period and follow-up time (ie, time since a first HIV-negative test), using clustered robust SEs to account for within-site correlation. We assessed correlation between follow-up time and mean testing frequency by calculating correlation coefficients for participants with more than two HIV tests. We calculated seroconversion rates for each demographic and behavioural risk factor. For time-varying factors, we used the outcome at the last recorded HIV-negative test for each participant (ie, the penultimate visit for women who seroconverted and the final test for those who remained HIV negative). To explore temporal trends in seroconversion, we used Poisson regression to estimate rate ratios and compared rates by study periods. We also estimated rate ratios for all other variables. We adjusted our model of seroconversion rates for age and HIV testing frequency to account for the changing demographic and testing patterns of people accessing services over time.

We did sensitivity analyses to assess our analytical decisions about cohort inclusion and seroconversion date estimation. In exploratory analysis, follow-up time was a strong predictor of seroconversion. Thus, we first restricted follow-up for each participant to a maximum of 2 years. We subsequently ran our analysis three more times, each time applying a different approach to estimating seroconversion dates. First, we used 1 month before a positive test as the seroconversion date to address the possibility that attending Sisters for HIV testing could be motivated by potential risk of exposure. Second, we used 2 weeks after a last negative test to address the potential that women had already been exposed at that time but had tested too early. Finally, we randomly assigned seroconversion dates by using the mean of 100 random runs of estimated seroconversion dates. We used Poisson regression to estimate rate ratios and compare seroconversion rates by time and adjusted our models as in our main analysis. We used Stata (version 17.0) for all statistical analyses.

### Role of the funding source

The funders of the study had no role in study design, data collection, data analysis, data interpretation, or writing of the report.

## Results

**Figure 1:**
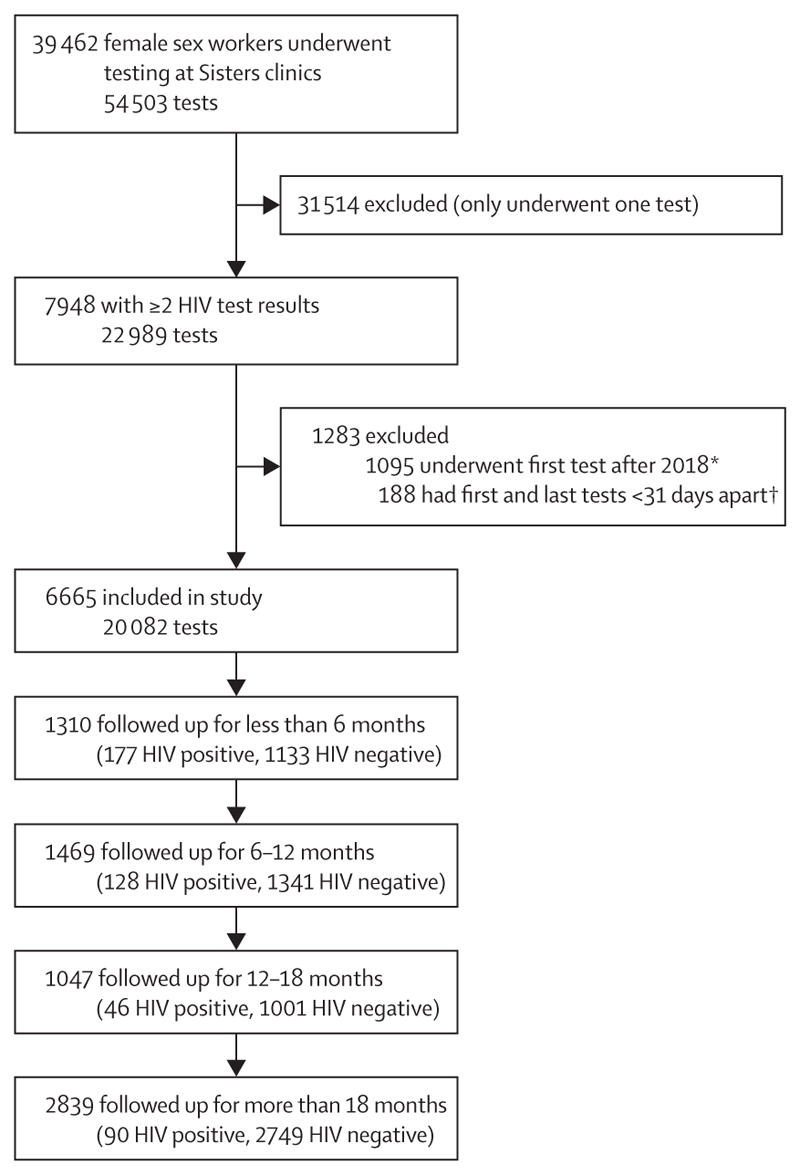
Flow diagram of cohort inclusion and follow-up Follow-up was defined as the time from the date of participants’ first HIV test to seroconversion (or date of last negative test if no positive HIV test was recorded). *35 of 2462 HIV tests were positive. †27 of 386 HIV tests were positive; a further 59 tests were excluded because they were done less than 7 days after the previous test (two of these were positive, and the previous negative result was excluded).

Between Sept 15, 2009, and Dec 31, 2019, 39 462 female sex workers underwent 54 503 HIV tests at a Sisters clinic. However, 31 514 had only one HIV test (8456 [27%] of which were positive) and were excluded from further analysis. A further 1283 participants with an initial HIV-negative test and at least one subsequent HIV test were excluded (accounting for 2907 tests; figure 1). Our analysis cohort thus comprised 6665 female sex workers, who underwent 20 082 HIV tests.

Median age at first HIV test in the analysis cohort was 27 years (IQR 23–32). The median age of women who were excluded because they underwent only one HIV test was 26 years (22–33). Among these women, median age was 25 years (21–32) among those who tested negative for HIV and 28 years (24–34) for those who tested positive. Other demographic characteristics and risk factors were similar between women who tested for HIV only once (and were thus excluded from our analysis) and those in the analysis cohort (appendix p 1).

During the study, 441 (7%) participants seroconverted. Median time between the first and last test was 409 days (IQR 222–702) among women who seroconverted compared with 476 days (239–896) among those who remained HIV negative. Both those who seroconverted and those who remained HIV negative did a median of two tests (IQR 2–4). Overall, the median between HIV tests was 266 days (159–452). In all study periods, education and marital status were similar between those who seroconverted and those who did not (table 1). 86 (20%) of the 441 women who seroconverted had less than 6 months between their first and last HIV test, compared with 1133 (18%) of the 6224 who remained HIV negative throughout the study. Overall, women who seroconverted were less likely to have more than 18 months between their first and last HIV test (160 [36%] *vs* 2749 [44%]) and were younger at first HIV test (median 25 years [IQR 22–31] *vs* 27 years [23–32]) than those who did not seroconvert (table 1).

**Figure 2:**
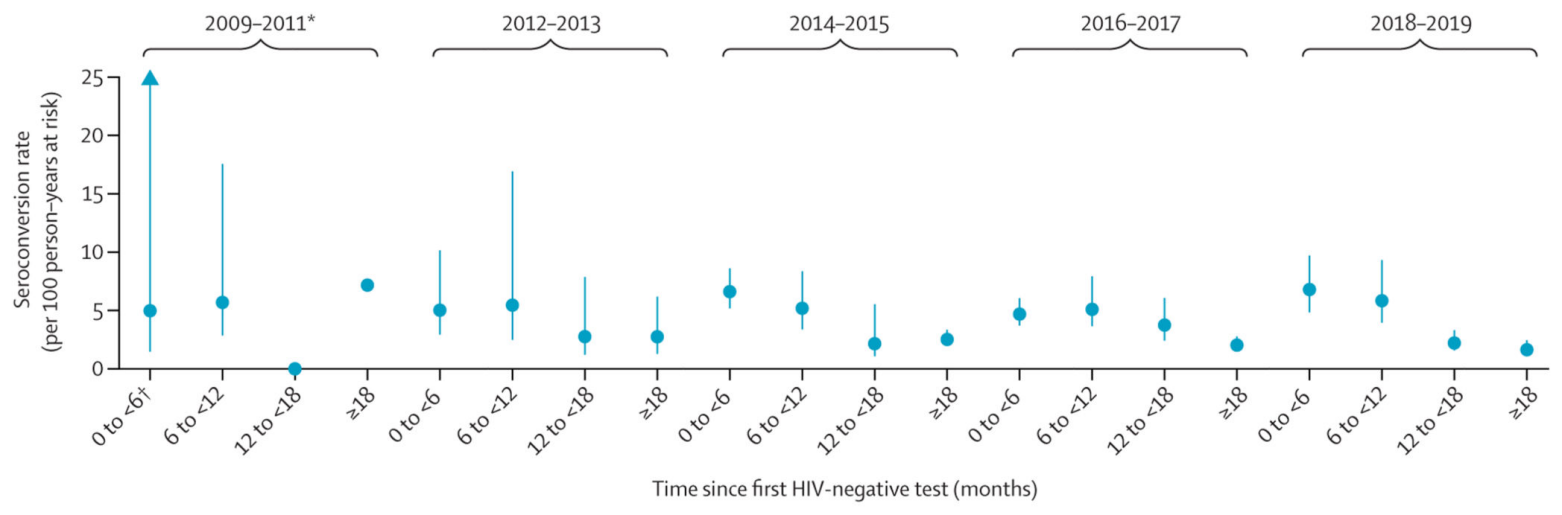
HIV seroconversion rates among female sex workers (n=6665) accessing Zimbabwe’s Sisters programme, by time since first HIV test Rates are shown for 2-year periods between 2009 and 2019: 2009–11 (nine seroconversions and 195 person-years at risk), 2012–13 (27 seroconversions and 674 person-years at risk), 2014–15 (95 seroconversions and 2132 person-years at risk), 2016–17 (152 seroconversions and 4283 person-years at risk), and 2018–19 (158 seroconversions and 4374 person-years at risk). Error bars represent 95% CIs. *Data for 2009 are from Sept 15 to Dec 31 only; 95% CIs could not be calculated for the 13–18 months’ follow-up and the >18 months’ follow-up groups. †95% CI=1·5–56·3.

Women contributed 11 657 person-years at risk, and the overall seroconversion rate during the study was 3·8 (95% CI 3·4–4·2) per 100 person-years at risk. Seroconversion rates were highest within 12 months of a first HIV-negative test, and fell among women who were followed up for more than a year (table 2). The rate ratio for seroconversion at least 18 months after an HIV test compared with in the first 6 months after an HIV test was 0·34 (95% CI 0·27–0·44; table 2). Seroconversion rates fell from 4·6 per 100 person-years at risk in 2009–11 to 3·6 per 100 person-years at risk in 2018–2019, but this difference was not significant in unadjusted analyses (table 2). Rates of seroconversion by follow-up time were similar in each 2-year study period (figure 2).

For 3504 (53%) participants, the mean time between HIV tests was the same as the length of follow-up because they underwent only two tests. The mean time between tests was correlated with follow-up time among women who underwent more than two tests (0·46; p<0·0001). Seroconversion rates were higher among women with a mean of less than 6 months between HIV tests than among those with a mean of more than 18 months between tests (rate ratio 0·29 [95% 0·23–0·37]; table 2).

In adjusted analyses, seroconversion rates were higher among participants younger than 25 years than those aged 25 years or older, among those with a syndromic STI diagnosis at their previous visit than among those without an STI diagnosis (table 2), and among those who reported using a condom during their most recent sexual encounter than among those who reported not using condoms (table 2). Seroconversion rates did not differ significantly between urban and rural sites or static and mobile sites, or between women who had ever experienced gender-based violence and those who had not (table 2).

When our model was adjusted for age and mean time between HIV tests, the risk of seroconversion was greater in 2009–11, 2012–13, and 2014–15 than in 2018–19 (table 2), and overall the risk of seroconversion between 2009 and 2019 decreased (p=0·0053). After adjustment for age and time between HIV tests, when seroconversion dates were randomly generated, seroconversion rates fell with time (p=0·0093; table 3). When 1 month before seroconversion was used as the seroconversion date, seroconversion rates fell between 2009 and 2017, but were highest in 2018–19 (table 3). In this sensitivity analysis, seroconversion rates were associated with calendar time in our adjusted model (p=0·0007). When 2 weeks after the last negative test result was used as the seroconversion date, seroconversion rates were higher between 2009 and 2015 than when either midpoint or random estimation were used to ascertain the seroconversion date (table 3). By 2018–19, seroconversion had fallen to 2·6 per 100 person-years at risk, with a strong downward trend in both crude and adjusted models (p<0·0001; table 3).

When follow-up time was restricted to 2 years, 6665 participants contributed 8268·9 person-years at risk and 386 seroconverted (rate 4·7 per 100 person-years at risk). Seroconversion rates in this restricted cohort showed little variation over time (table 3).

## Discussion

In our study, seroconversion rates were high among female sex workers accessing HIV services through Zimbabwe’s Sisters programme, but there was evidence of a steady decline over time after adjustment for age and individual HIV testing frequency. Seroconversion rates were higher within 6 months of a first HIV-negative test, among those testing more frequently, among those younger than 25 years, and among those diagnosed with an STI at a previous visit. Our findings were generally robust to sensitivity analyses, but when we simulated testing strongly motivated by recent risk (by using 1 month before a negative test as the seroconversion date), seroconversion rates no longer decreased over time.

Seroconversion rates in our study were similar to those previously reported for female sex workers accessing HIV services through the Sisters programme.^13,14^ Rates for individual periods reflect those reported in other studies of female sex workers^15–17^ in southern Africa between 2009 and 2019, although the rate for 2018–19 in our study was lower than the 4·6 per 100 person-years at risk reported in South Africa for 2019.^18^ The decline in seroconversion rates over time that we report reflects both modelled and empirical estimates reported for the general population in southern Africa.^11,19,20^ We identified variables commonly associated with increased risk of HIV infection, including younger age and diagnosis with an STI.^21^

Our findings are consistent with the age-specific prevalence of HIV reported among female sex workers recruited for respondent-driven sampling surveys in Zimbabwe,^16^ in which the incidence of HIV was higher among female sex workers aged 18–24 years than among those aged 25–39 years (6·3 per 100 person-years at risk compared with 3·3 per 100 person-years at risk). A similar incidence of 5·3 per 100 person-years at risk was also reported for young women selling sex in non-intervention sites in the DREAMS study.^17^ Studies suggest a higher frequency of new HIV infections before formal entry into sex work^22^ compared with already being a sex worker and soon after first selling sex compared with having been a sex worker for longer,^16^ aligning with the higher seroconversion rates we reported close to first testing for HIV at a Sisters clinic. Although a first HIV test could be a proxy for recent entry into sex work, we could have underestimated incidence by excluding the period before formal entry into sex work and engagement with the Sisters programme and prevention services.^23^ Our data give some indication of this potential underestimation, with 27% of tests positive among female sex workers with only one Sisters HIV test result. Given the median age of 27 years at a first test in our study, and that access to Sisters services requires self-identification as a sex worker, it is unlikely that our findings fully account for this period of recent entry into sex work before self-identification as a sex worker.

A key strength of our study was the large programme dataset, which enabled analysis of HIV test data for 6665 female sex workers over 10 years. As a result, we could report temporal trends in the incidence of new HIV infections, which have not been previously reported for female sex workers in southern Africa.^10^ Our study had several limitations. The generalisability of our findings could be limited by the decision to include only sex workers who accessed Sisters clinic services and returned for repeat HIV testing. However, with programme coverage increasing substantially over time through expanded community outreach and clinic referral and the use of peer educators and networks within the sex worker community, our cohort was probably increasingly representative of female sex workers in Zimbabwe. In 2017, the programme’s clinical services were thought to have reached 57% of the estimated 40 000 female sex workers in the country,^24^ and our findings are similar to those reported in respondent-driven sampling surveys, which reached female sex workers who had not accessed Sisters services. Female sex workers excluded from our study because they had only one HIV test result available were slightly younger than those included in the analysis, but were similarly distributed across clinic sites and had similar demographic characteristics. Our findings might be less generalisable to women and young girls who do not identify as sex workers. Although we included time-varying risk factors in our analysis, we could not do this for all variables, which limited our ability to adjust for confounding by variables that were measured only at the first clinic visit. We used proximity to a last HIV-negative test for STI diagnoses and condom use, but variation in time between HIV tests meant that proximity varied for individual women, and could have greater relevance when measured closer to HIV-positive test results. Our analysis did not include variables for which data were not collected, including time in sex work and HIV test refusal, or variables for which over 40% of data were missing, including number of condomless sex partners, which could have helped with interpretation. Our analysis pre-dated widespread rollout of pre-exposure prophylaxis in Zimbabwe.

Identification of the time of HIV infection at diagnosis is challenging.^25^ In our study, imputation of seroconversion dates either at the midpoint between testing positive for HIV and the last negative test result or randomly gave similar results, as has been reported elsewhere.^17,26^ Unlike more conventional cohorts that have set follow-up times, the continuous enrolment of women in our cohort means that midpoint estimation is unlikely to have caused clustering of seroconversions around the middle of the reporting period, and therefore is unlikely to have suggested inaccurate declines in seroconversion frequency towards the end of the study.^27^ However, despite the exclusion of women testing for the first time after 2018, seroconversion rates at the end of our reporting period could have been inflated if HIV test data were not available for women who later returned to the programme.^13,14^ In other populations, studies have shown that HIV infection is not independent of testing patterns in public health testing services^28^ and have suggested that seroconversion occurs closer to an HIV-positive test than midpoint estimation sometimes suggests.^25^ In sensitivity analyses in our study, use of 1 month before testing positive for HIV as the seroconversion suggested that seroconversion rates were not declining with time. However, female sex workers are recommended to test more frequently than non-sex workers, which makes the relevance of assumptions about risk exposure driving testing less clear.

Our study identified the effect of wide variation in follow-up time and time between HIV tests on our findings. As follow-up time and time between tests were correlated, we chose to adjust for mean time between HIV tests as an indicator of testing engagement and reduced certainty around seroconversion dates with longer time between tests. Regular HIV testing could indicate high-risk behaviour and potential HIV exposure. Testing patterns among female sex workers accessing Sisters have changed over time,^13^ as have testing guidelines,^29^ and the availability and acceptability of testing have increased. HIV-exposure-driven testing might have become more common with expansion of the Sisters programme. Our model adjusted for increased testing frequency and age showed evidence of decreasing seroconversion rates over time by accounting for potential confounding caused by increased testing and for bias introduced by early testing among individuals with recent infections.^30^ We restricted follow-up to 2 years in a sensitivity analysis to reduce potential bias introduced by the disproportionate contribution of HIV-negative follow-up from a small group of long-term engaged participants. The results of this analysis suggest that there are potential biases in our approach, and that our results are sensitive to analytical choices for estimation of seroconversion rates over shorter periods. Restriction of follow-up to 2 years increased seroconversion rates in later periods. Our overall interpretation of falling seroconversion rates over time remained, although this sensitivity analysis shows that inclusion of all possible follow-up time could underestimate seroconversion rates in later periods.

Our study suggests that, although seroconversion rates remain consistently high among female sex workers accessing services, seroconversion risk has decreased overall. Through peer outreach, Sisters has worked to reach women and girls at high risk of acquiring HIV over time; the proportion of younger women accessing Sisters services has increased, as have engagement with services and HIV testing frequency. Although our study supports previous evidence that younger women are at increased risk of HIV infection, we also found that women are twice as likely to seroconvert within 6 months of first testing compared with those whose first test was more than 18 months ago. This finding suggests that women at higher risk for HIV, potentially new to sex work, are being reached, but need to be engaged in prevention interventions earlier—ie, before or as they transition into sex work—to maximise the effect of interventions on new infections.

Our findings have implications for the delivery of HIV testing for female sex workers. Increasing the recommended testing frequency to every 3 months for the first year of programme engagement could be an important intervention during this period of increased seroconversion risk. For women who remain HIV negative after a year, testing frequency could be reduced in line with published guidelines. Future analysis should include data for initiation of sex work to account for the period of transition into sex work and early sex work. WHO has advocated for better use of routinely collected data to inform programming,^31^ and risk factors identified in this study could be used to provide more intensive and targeted support to specific groups of female sex workers, as is done with risk-differentiated microplanning.^32^

New HIV infections remain challenging to measure, although longitudinal analysis of routine HIV testing data with linkage of individuals over time can provide valuable insights into seroconversion rates and associated risk factors. Our findings highlight the potential to draw biased conclusions when estimating HIV incidence from routine data as a result of testing patterns, and thus there is a need to strengthen HIV surveillance approaches among female sex workers and other key populations. Our findings also show the continued need to intensify HIV prevention among female sex workers in Zimbabwe, given the high rates of seroconversion identified. Both measurement approaches and programming need to be strengthened to reduce new HIV infections and achieve sustainable epidemic control.

## Supplementary Material

Supplementary appendix

## Figures and Tables

**Table 1:** Characteristics of repeat HIV testers in the Sisters programme, by seroconversion status and time

	2009–11 (n=239)		2012–13 (n=431)		2014–15(n=1745)		2016–17 (n=2639)		2018–19 (n=1611)	
	HIV negative (n=209)	HIV positive (n=30)	HIV negative (n=397)	HIV positive (n=34)	HIV negative (n=1604)	HIV positive (n=141)	HIV negative (n=2479)	HIV positive (n=160)	HIV negative (n=1535)	HIV positive (n=76)
Site location										
Urban	150 (72%)	24 (80%)	371 (93%)	31 (91%)	1123 (70%)	105 (74%)	2044 (82%)	142 (89%)	1398 (91%)	71 (93%)
Rural	59 (28%)	6 (20%)	26 (7%)	3 (9%)	481 (30%)	36 (26%)	435 (18%)	18 (11%)	137 (9%)	5 (7%)
Site type										
Static	128 (61%)	19 (63%)	283 (71%)	27 (79%)	718 (45%)	76 (54%)	1880 (76%)	121 (76%)	1363 (89%)	67 (88%)
Mobile	81 (39%)	11 (37%)	114 (29%)	7 (21%)	886 (55%)	65 (46%)	599 (24%)	39 (24%)	172 (11%)	9 (12%)
Median age at first HIV test, years	29 (24–36)	25 (22–31)	29 (24–33)	26 (23–30)	28 (23–34)	24 (21–31)	26 (22–32)	26 (21–31)	26 (22–31)	26 (22–30)
Education										
None or primary	20 (30%)	1 (7%)	84 (22%)	8 (25%)	390 (25%)	39 (28%)	412 (18%)	25 (16%)	244 (17%)	13 (18%)
Secondary or tertiary	46 (70%)	13 (93%)	292 (78%)	24 (75%)	1170 (75%)	99 (72%)	1906 (82%)	127 (84%)	1180 (83%)	60 (82%)
Missing	143	16	21	2	44	3	161	8	111	3
Marital status										
Married	1 (1<%)	0 (0)	14 (4%)	0 (0)	44 (3%)	1 (1%)	63 (3%)	3 (2%)	59 (4%)	1 (1%)
Divorced	134 (64%)	22 (73%)	231 (59%)	24 (71%)	1094 (69%)	96 (70%)	1518 (65%)	102 (67%)	876 (61%)	47 (64%)
Never married	36 (17%)	8 (27%)	87 (22%)	7 (21%)	285 (18%)	26 (19%)	604 (26%)	41 (27%)	442 (31%)	21 (29%)
Separated	3 (1%)	0 (0)	29 (7%)	1 (3%)	20 (1%)	2 (1%)	4 (<1%)	0 (0)	2 (<1%)	0 (0)
Widowed	34 (16%)	0 (0)	33 (8%)	2 (6%)	135 (9%)	13 (9%)	142 (6%)	6 (4%)	48 (3%)	4 (5%)
Missing	1	0	3	0	26	3	148	8	108	3
Condom used with most recent sexual partner										
No	95 (53%)	7 (39%)	224 (59%)	15 (47%)	1024 (66%)	82 (59%)	1758 (75%)	120 (75%)	1088 (75%)	53 (75%)
Yes	84 (47%)	11 (61%)	155 (41%)	17 (53%)	533 (34%)	58 (41%)	596 (25%)	39 (25%)	361 (25%)	18 (25%)
Missing	30	12	18	2	47	1	125	1	86	5
STI diagnosis*										
No	134 (64%)	20 (67%)	267 (67%)	20 (59%)	1146 (71%)	77 (55%)	1749 (71%)	85 (53%)	1138 (74%)	45 (59%)
Yes	75 (36%)	10 (33%)	130 (33%)	14 (41%)	458 (29%)	64 (45%)	730 (29%)	75 (47%)	397 (26%)	31 (41%)
Gender-based violence (ever)										
No	188 (90%)	25 (83%)	278 (70%)	23 (68%)	1149 (73%)	103 (74%)	1882 (81%)	121 (80%)	1231 (87%)	57 (79%)
Yes	21 (10%)	5 (17%)	119 (30%)	11 (32%)	432 (27%)	36 (26%)	436 (19%)	30 (20%)	187 (13%)	15 (21%)
Missing	0	0	0	0	23	2	161	9	117	4
HIV tests										
Median number	3 (2–4)	2 (2–3)	3 (2–5)	2 (2–3)	3 (2–4)	2 (2–3)	2 (2–3)	2 (2–3)	2 (2–3)	2 (2–3)
Median time between last two tests	426 (210–1134)	365 (182–761)	308 (157–736)	477 (247–1086)	305 (146–651)	301 (155–652)	246 (122–470)	298 (176–546)	175 (101–280)	203 (86–351)
Time between first and last HIV test										
0 to <6 months	15 (7%)	4 (13%)	35 (9%)	3 (9%)	219 (14%)	28 (20%)	427 (17%)	26 (16%)	437 (28%)	25 (33%)
6 to <12 months	18 (9%)	2 (7%)	48 (12%)	7 (21%)	220 (14%)	27 (19%)	442 (18%)	43 (27%)	613 (40%)	27 (36%)
12 to <18 months	15 (7%)	7 (23%)	32 (8%)	2 (6%)	170 (11%)	23 (16%)	392 (16%)	35 (22%)	392 (26%)	22 (29%)
≥18 months	161 (77%)	17 (57%)	282 (71%)	22 (65%)	995 (62%)	63 (45%)	1218 (49%)	56 (35%)	93 (6%)	2 (3%)
Mean testing frequency										
0 to <6 months	17 (8%)	4 (13%)	61 (15%)	4 (12%)	372 (23%)	35 (25%)	743 (30%)	41 (26%)	804 (52%)	35 (46%)
6 to <12 months	40 (19%)	9 (30%)	114 (29%)	12 (35%)	538 (34%)	46 (33%)	905 (37%)	48 (30%)	551 (36%)	25 (33%)
12 to <18 months	42 (20%)	5 (17%)	70 (18%)	5 (15%)	268 (17%)	22 (16%)	399 (16%)	39 (24%)	158 (10%)	16 (21%)
≥18 months	110 (53%)	12 (40%)	152 (38%)	13 (38%)	426 (27%)	38 (27%)	432 (17%)	32 (20%)	22 (1%)	0 (0)

Data are n (%), median (IQR), or n. For percentage calculations, we used available data as the denominator rather than the total N. STI=sexually transmitted infection. *Diagnosed with an STI at the last recorded HIV-negative test (the penultimate visit for women who seroconverted and the final test for those who remained HIV negative).

**Table 2:** Seroconversion rates by demographic and HIV testing characteristics (n=441)

	Seroconversions	Person-years at risk/100	Seroconversion rate per 100 person-years (95% CI)	Rate ratio (95% CI)	Adjusted rate ratio* (95% CI)	p value
Period	··	··	··	··	··	0·0053
2009–11	9	1·9	4·6 (2·6–12·7)	1·28 (0·80–2·04)	1·78 (1·15–2·75)	··
2012–13	27	6·7	4·0 (2·8–6·3)	1·11 (0·72–1·70)	1·61 (1·02–2·55)	··
2014–15	95	21·3	4·5 (3·5–5·7)	1·23 (0·93–1·63)	1·54 (1·20–1·97)	··
2016–17	152	42·8	3·5 (3·1–4·1)	0·98 (0·79–1·23)	1·15 (0·92–1·43)	··
2018–19	158	43·7	3·6 (2·9–4·6)	1 (ref)	1 (ref)	··
Follow-up time†	··	··	··	··	··	<0·0001
0 to <6 months	177	30·7	5·8 (5·0–6·7)	1 (ref)	1 (ref)	··
6 to <12 months	128	23·1	5·5 (4·6–6·5)	0·95 (0·80–1·14)	0·94 (0·76–1·16)	··
12 to <18 months	46	16·6	2·8 (2·1–3·7)	0·48 (0·33–0·71)	0·50 (0·33–0·75)	··
≥18 months	90	46·1	2·0 (1·6–2·4)	0·34 (0·27–0·44)	0·37 (0·28–0·51)	··
Mean time between tests	··	··	··	··	··	<0·0001
0 to <6 months	119	15·1	7·9 (6·8–9·3)	1 (ref)	1 (ref)	··
6 to <12 months	140	37·8	3·7 (3·2–4·4)	0·47 (0·37–0·60)	0·50 (0·39–0·63)	··
12 <18 months	87	22·9	3·8 (2·8–5·3)	0·48 (0·36–0·63)	0·49 (0·38–0·64)	··
≥18 months	95	40·8	2·3 (1·8–3·0)	0·29 (0·23–0·37)	0·31 (0·23–0·40)	··
Site location	··	··	··	··	··	··
Urban	373	92·9	4·0 (3·6–4·5)	1 (ref)	1 (ref)	··
Rural	68	23·7	2·9 (2·3–3·6)	0·72 (0·57–0·91)	0·81 (0·64–1·02)	0·067
Site type	··	··	··	··	··	··
Static	310	77·0	4·0 (3·6–4·5)	1 (ref)	1 (ref)	··
Mobile	131	39·6	3·3 (2·7–4·0)	0·82 (0·66–1·01)	0·93 (0·76–1·14)	0·48
Age	··	··	··	··	··	··
<25 years	177	31·7	5·6 (4·8–6·5)	1 (ref)	1 (ref)	··
≥25 years	251	81·8	3·1 (2·7–3·5)	0·55 (0·45–0·67)	0·59 (0·48–0·73)	<0·0001
Education	··	··	··	··	··	··
None or primary	86	22·3	3·9 (3·2–4·7)	1·03 (0·82–1·29)	0·96 (0·76–1·22)	0·73
Secondary or tertiary	323	81·7	4·0 (3·5–4·6)	1 (ref)	1 (ref)	··
Marital status	··	··	··	··	··	0·0044
Married	5	3·2	1·6 (0·4–23·2)	0·40 (0·17–0·95)	0·38 (0·16–0·90)	··
Divorced	291	73·3	4·0 (3·4–4·7)	1 (ref)	1 (ref)	··
Never married	103	25·2	4·1 (3·6–4·7)	1·03 (0·82–1·29)	0·86 (0·68–1·08)	··
Separated	3	1·7	1·8 (0·6–7·0)	0·46 (0·15–1·35)	0·53 (0·17–1·63)	··
Widowed	25	9·4	2·7 (1·8–4·0)	0·67 (0·45–1·01)	0·84 (0·56–1·26)	··
Condom used with most recent sexual partner	··	··	··	··	··	··
No	277	79·9	3·5 (3·0–4·1)	1 (ref)	1 (ref)	··
Yes	143	31·1	4·6 (4·0–5·2)	1·33 (1·06–1·65)	1·33 (1·07–1·64)	0·0086
STI diagnosis‡	··	··	··	··	··	··
No	247	84·4	2·9 (2·6–3·3)	1 (ref)	1 (ref)	··
Yes	194	32·2	6·0 (5·3–7·0)	2·06 (1·84–2·30)	2·16 (1·97–2·35)	<0·0001
Gender-based violence (ever)	··	··	··	··	··	··
No	329	87·3	3·8 (3·3–4·4)	1 (ref)	1 (ref)	··
Yes	97	25·4	3·8 (3·3–4·6)	1·02 (0·81–1·27)	1·08 (0·87–1·35)	0·47
Time from last negative test to seroconversion	··	··	··	··	··	··
≤365 days	261	60·6	4·3 (3·8–4·9)	1 (ref)	1 (ref)	··
>365 days	180	55·9	3·2 (2·8–3·8)	0·75 (0·64–0·88)	1·50 (1·16–1·94)	0·0021

STI=sexually transmitted infection. *Adjusted for age and mean time between HIV tests. †Time from first programme test to midpoint estimated seroconversion date (or date of last HIV-negative test date if no HIV-positive result). ‡Diagnosed with an STI at the last recorded HIV-negative test for each woman (the penultimate visit for women who seroconverted and the final test for those who remained HIV-negative).

**Table 3:** Seroconversion rates by method of estimation of seroconversion date (n=6665)

	Overall cohort							Cohort restricted to 2 years’ follow-up				
	Seroconversions	Person-years at risk/100	Seroconversion rate per 100 person-years (95% CI)	Rate ratio (95% Cl)	Adjusted rate ratio* (95% CI)	p value		Séroconversions	Person-years at risk/100	Seroconversion rate per 100 person-years (95% CI)	Adjusted rate ratio* (95% CI)	p value
Midpoint	··	··	··	··	··	0·0053		··	··	··	··	0·054
2009–11	9	1·9	4·6 (2·6–12·7)	1·30 (0·82–2·04)	1·78 (1·15–2·75)	··		9	1·9	4·6 (2·7–12·7)	1·57 (0·98–2·50)	··
2012–13	27	6·7	4·0 (2·8–6·3)	1·13 (0·73–1·73)	1·61 (1·02–2·55)	··		24	5·4	4·4 (2·9–7·4)	1·51 (0·88–2·60)	··
2014–15	95	21·3	4·5 (3·5–5·7)	1·26 (0·95–1·67)	1·54 (1·20–1·97)	··		88	16·7	5·3 (43–6·6)	1·44 (1·11–1·87)	··
2016–17	152	42·8	3·5 (3·1–4·1)	0·99 (0·80–1·23)	1·15 (0·92–1·43)	··		130	30·5	4·3 (3·5–5·5)	1·06 (0·79–1·43)	··
2018–19	158	43·7	3·6 (2·9–4·6)	1 (ref)	1 (ref)	··		135	28·2	4·8 (3·8–6·1)	1 (ref)	··
1 month before seroconversion	··	··	··	··	··	0·0007		··	··	··	··	0·0002
2009–11	8	2·0	4·1 (1·7–20·7)	0·84 (0·49–1·46)	141 (0·65–1·89)	··		8	2·0	4·1 (1·8–20·7)	1·64 (1·06–2·52)	··
2012–13	21	6·8	3·1 (1·7–6·3)	0·64 (0·35–1·19)	0·87 (0·45–1·70)	··		17	5·5	3·1 (1·5–8·4)	1·25 (0·54–2·91)	··
2014–15	71	21·7	3·3 (2·4–4·5)	0·68 (0·49–0·96)	0·81 (0·59–1·11)	··		61	16·9	3·6 (2·6–5·2)	1·02 (0·73–1·41)	··
2016–17	127	43·6	2·9 (2·4–3·6)	0·61 (0·45–0·83)	0·68 (0·50–0·94)	··		101	30·9	3·3 (2·6–4·2)	0·83 (0·58–1·18)	··
2018–19	214	44·6	4·8 (3·9–5·9)	1 (ref)	1 (ref)	··		149	28·7	5·2 (41–6·6)	1 (ref)	··
2 weeks after last negative test	··	··	··	··	··	<0·0001		··	··	··	··	<0·0001
2009–11	20	1·9	10·8 (8·1–14·2)	4·20 (2·76–6·40)	5·98 (3·84–9·32)	··		20	1·8	10·8 (8·1–14·2)	4·43 (2·53–7·76)	··
2012–13	34	6·5	5·3 (4·l–7·4)	2·05 (1·41–2·98)	3·06 (2·02–4·63)	··		34	5·2	6·6 (53–9·0)	2·71 (1·65–4·44)	··
2014–15	122	20·7	5·9 (4·4–7·8)	2·30 (1·58–3·36)	2·85 (2·04–3·99)	··		119	16·1	7·4 (6·0–9·l)	2·58 (1·75–3·79)	··
2016–17	154	41·9	3·7 (3·0–4·6)	1·43 (1·11–1·86)	1·62 (1·23–2·14)	··		146	29·6	4·9 (3·9–6·5)	1·55 (1·08–2·23)	··
2018–19	111	43·3	2·6 (1·9–3·6)	1 (ref)	1 (ref)	··		95	27·8	3·4 (2·5–4·9)	1 (ref)	··
Random	··	··	··	··	··	0·0093		··	··	··	··	0·069
2009–11	9	1·9	4·6 (2·6–12·6)	1·27 (0·79–2·04)	1·77 (1·13–2·76)	··		9	1·9	4·6 (2·7–12·7)	1·55 (0·97–2·48)	··
2012–13	27	6·7	4·0 (2·5–6·3)	1·10 (0·72–1·68)	1·60 (1·02–2·52)	··		24	5·4	4·4 (3·0–7·4)	1·49 (0·87–2·57)	··
2014–15	96	21·3	4·5 (3·5–5·8)	1·24 (0·94–1·63)	1·54 (1·21–1·97)	··		86	16·7	5·2 (4·1–6·5)	1·45 (1·12–1·90)	··
2016–17	151	42·8	3·5 (3·1–4·2)	0·96 (0·78–1·18)	1·13 (0·92–1·38)	··		127	30·5	4·2 (3·3–5·5)	1·07 (0·79–1·45)	··
2018–19	158	43·7	3·6 (3·0–4·5)	1 (ref)	1 (ref)	··		136	28·2	4·8 (3·9–6·0)	1 (ref)	··

*Adjusted for age and mean time between HIV tests; 6414 people were included in adjusted models because data were missing for age for the other 251 participants.

## Data Availability

A de-identified dataset with variables included in this analysis can be made available on request to the Centre for Sexual Health & HIV/AIDS Research Zimbabwe, subject to ethical approval of a proposal.

## Research in context

### Evidence before this study

We searched MEDLINE, EMBASE, POPLINE, Web of Science, and Global Health with the medical subject headings and text words “female sex worker”, “HIV”, and “sub-Saharan Africa” for articles published in English between Jan 1, 1990, and June 4, 2019. We updated our search to include articles published up to Dec 15, 2022, and also searched unpublished literature for a review of estimates of HIV incidence among female sex workers in sub-Saharan Africa. Estimates of incidence ranging from 0·4 per 100 person-years to 42 per 100 person-years were identified for 31 independent study populations of female sex workers in 14 countries between 1987 and 2020. Very few studies were nationally representative and inclusion criteria, recruitment methods, and analytical approaches varied widely. As a result, comparison of estimates and pooling of data is challenging. Only two study populations provided estimates from more than one timepoint, making temporal trends difficult to ascertain.

### Added value of this study

This study—which is based on data from Sisters with a Voice, a nationally representative sex worker programme in Zimbabwe—contributes new estimates of the frequency of new HIV infections among female sex workers to the scarce empirical data available for this population in sub-Saharan Africa. The comprehensive longitudinal dataset provided by Sisters enabled analysis of seroconversion rates in the same population over 10 years, which has not been possible elsewhere in the region. This study additionally showed the methodological limitations of the use of routine data for analyses of HIV incidence and provided a nuanced overview of the effect of the various analytical decisions that need to be made to draw robust conclusions from these data.

### Implications of all the available evidence

Among female sex workers accessing sexual and reproductive health services from Sisters in Zimbabwe, rates of HIV seroconversion remain high, but there is evidence of a steady decline over time. High seroconversion rates soon after initial service engagement are consistent with previous evidence of increased acquisition risk before and during formal entry into sex work, suggesting the need for earlier intervention and enhanced HIV prevention services from first contact. As HIV surveillance transitions to routine-dominated surveillance, the development of methods to estimate the frequency of new HIV infections in key populations from programme data is becoming increasingly important—particularly approaches that address challenges in follow-up and that provide insight into new infections among people who do not access services. Empirical estimates of new HIV infections are crucial to tracking and ensuring future progress in the HIV response to achieve sustainable epidemic control.
