# Supplementary material for: Temporal trends in, and risk factors for, HIV seroconversion among female sex workers accessing Zimbabwe’s national sex worker programme, 2009–19: a retrospective cohort analysis of routinely collected HIV testing data
Source: Lancet HIV. Author manuscript; Available in PMC 2025 Oct 1. (PMC7618186; doi:10.1016/S2352-3018(23)00110-8)
Supplement: Supplementary appendix [file EMS208729-supplement-Supplementary_appendix.pdf]

THE LANCET
HIV

Supplementary appendix

This appendix formed part of the original submission and has been peer reviewed. We post it as supplied by the authors.

Supplement to: Jones HS, Hensen B, Musemburi S, et al. Temporal trends in, and risk factors for, HIV seroconversion among female sex workers accessing Zimbabwe's national sex worker programme, 2009–19: a retrospective cohort analysis of routinely collected HIV testing data. *Lancet HIV* 2023; published online June 14. https://doi.org/10.1016/S2352-3018(23)00110-8.

Appendix 1: Characteristics of female sex workers with a single HIV test compared to repeat HIV testers at Sisters with a Voice clinics in Zimbabwe between 2009 and 2019

	**Included**			**Excluded (1 HIV test)**		
	(n=6665)			(n=31,514)		
	**All women** (n=6665)	**HIV-negative** (n=6624)	**Seroconversions** (n=441)	**All women** (n=31,514)	**HIV-negative** (n=23,058)	**HIV-positive** (n=8,456)
**Calendar year**						
2009-2011	239 (2.0)	230 (2.0)	9 (2.0)	1586 (5.1)	425 (1.8)	1161 (13.7)
2012-2013	628 (5.3)	601 (5.3)	27 (6.1)	1304 (4.1)	560 (2.4)	744 (8.8)
2014-2015	2234 (18.9)	2139 (18.7)	95 (21.5)	5021 (15.9)	3015 (13.1)	2006 (23.7)
2016-2017	4310 (36.4)	4158 (36.4)	152 (34.5)	8472 (26.9)	6268 (27.2)	2204 (26.1)
2018-2019	4443 (37.5)	4285 (37.5)	158 (35.8)	15131 (48.0)	12790 (55.5)	2341 (27.7)
**Site location**						
Urban	5459 (81.9)	5086 (81.7)	373 (84.6)	26181 (83.1)	19183 (83.2)	6998 (82.8)
Rural	1206 (18.1)	1138 (18.3)	68 (15.4)	5333 (16.9)	3875 (16.8)	1458 (17.2)
**Site type**						
Static	4742 (71.2)	4425 (71.1)	317 (71.9)	21067 (66.9)	15425 (66.9)	5642 (66.7)
Mobile	1923 (28.9)	1799 (28.9)	124 (28.1)	10447 (33.2)	7633 (33.1)	2814 (33.3)
**Demographics**						
Median age at first test (IQR)	27 (23-32)	27 (23-32)	25 (22-31)	26 (22-33)	25 (21-32)	28 (24-34)
**Education**						
None/Primary	1236 (20.1)	1150 (20.0)	86 (21.0)	5698 (19.9)	3916 (18.3)	1782 (24.5)
Secondary/Tertiary	4,917 (79.9)	4594 (80.0)	323 (79.0)	22968 (80.2)	17490 (81.7)	5478 (75.5)
Missing	*512*	*480*	*32*	*2,848*	*1,652*	*1,196*
**Marital status**						
Currently married	186 (2.9)	181 (3.1)	5 (1.2)	822 (2.8)	659 (3.0)	163 (2.0)
Divorced	4144 (65.1)	3853 (64.9)	291 (68.2)	17921 (59.9)	12633 (58.1)	5288 (64.6)
Never married	1557 (24.5)	1454 (24.5)	103 (24.1)	8824 (29.5)	7149 (32.9)	1675 (20.5)
Separated	61 (1.0)	58 (1.0)	3 (0.7)	153 (0.5)	90 (0.4)	63 (0.8)
Widowed	417 (6.6)	392 (6.6)	25 (5.9)	2192 (7.3)	1200 (5.5)	992 (12.1)
missing	*300*	*286*	*14*	*1,602*	*1,327*	*275*
**Recent condom use**						
No	4466 (70.5)	4189 (70.8)	277 (66.0)	20110 (68.4)	15100 (69.5)	5010 (65.4)
Yes	1872 (29.5)	1729 (29.2)	143 (34.0)	9293 (31.6)	6637 (30.5)	2656 (34.6)
Missing	*327*	*306*	*21*	*2,111*	*1,321*	*790*
**STI**						
No	4681 (70.2)	4434 (71.2)	247 (56.0)	20435 (64.8)	15499 (67.2)	4936 (58.4)
Yes	1984 (29.8)	1790 (28.8)	194 (44.0)	11079 (35.2)	7559 (32.8)	3520 (41.6)
**Gender based violence**						
No	5057 (79.7)	*4728 (79.8)*	329 (77.2)	24903 (83.5)	18353 (84.7)	6550 (80.2)
Yes	1292 (20.3)	*1195 (20.2)*	97 (22.8)	4931 (16.5)	3316 (15.3)	1615 (19.8)
missing	*316*	*301*	*15*	*1,680*	*1,389*	*291*
